# Parent-Child Attachment: A Principle-Based Concept Analysis

**DOI:** 10.1177/23779608211009000

**Published:** 2021-06-16

**Authors:** E. Ali, N. Letourneau, K. Benzies

**Affiliations:** University of Calgary, Calgary, Alberta, Canada

**Keywords:** attachment, concept analysis, nursing practice

## Abstract

**Introduction:**

Extensive evidence indicates that the quality of parent-child attachment is related to later socio-emotional and physical health outcomes. Yet, despite its clinical relevance, the parent-child attachment concept has been inconsistently applied across the disciplines of nursing, medicine and psychology and is often conflated with parent-child bonding in nursing literature.

**Objectives:**

To provide readers with a critical analysis of the concept of parent-child attachment. Using a principle-based concept analysis, we clarify how parent-child attachment is understood from a multidisciplinary perspective to advance the use of this concept in nursing practice.

**Concept Description:** Attachment is an affectionate, mutually satisfying relationship between a child and a caregiver that serves the purpose of making the child feel safe, secure, and protected.

**Discussion:**

In this principle-based concept analysis, each definitional (i.e., epistemological, pragmatic, linguistic, and logical) principle contributes to an understanding of the strengths and limitations of the state of science about this concept. The discussion highlights how applying the concept of parent-child attachment security may offer exciting and promising opportunities for nursing clinical work with families.

**Conclusion:**

The understanding of the concept of parent-child attachment differs among disciplines of nursing, medicine and psychology and offers exciting and promising opportunities for clarity and collaborative, multi-disciplinary work.

## Introduction

Attachment is an affectionate, mutually satisfying relationship between a child and a caregiver (e.g., a parent) who is involved in making the child feel safe, secure, and protected (Bowlby, 1969/1982). According to attachment theory, a child who consistently experiences responsive and sensitive caregiving develops an expectation that others will be supportive and available in times of need (Fraley, 2002). The founder of attachment theory, British psychoanalyst John Bowlby (1944, 1951), studied how the child’s early experiences with the caregiver impacted the child’s future mental health. Based on his work with hospitalized and institutionalized children, Bowlby (1951) concluded that to thrive emotionally and grow up mentally healthy, a child must experience a mutually affectionate relationship with the primary caregiver. Bowlby contended that when distressed or alarmed, infants were disposed to attain closeness with their caregiver through signals and movements, such as crying, smiling, sucking, clinging, and crawling. This disposition towards a caregiver was described as an “attachment system”, with the actions of the infant being termed “attachment behavior” (Bowlby, 1969/1982). Bowlby identified the emotional and physical availability of the caregiver as a “set-goal” of attachment in infancy. According to Bowlby (1982), attachment behaviors are rooted in evolution and increase the likelihood of survival by increasing caregiver-child proximity.

The attachment system serves to keep the caregiver close to protect the infant in case of a physical or psychological threat (Bowlby, 1982). Bowlby claimed that a well-loved infant will protest separation from parents, but will later develop more self-reliance (Bretherton, 1992). In the first volume of his trilogy, *Attachment, and Loss*, Bowlby (1969) considered the antecedents of attachment, such as maternal sensitivity to an infant’s cues. Bowlby’s (1951) discovery of just how much children depend on their parents for support led to his famous quote that still rings true to this day: “if a community values its children it must cherish their parents” (p. 84). Bowlby’s collaboration with the Canadian psychologist Mary Ainsworth led to the development of attachment theory. Parent-child attachment (PCA) security is one of the factors in determining future socio-emotional functioning and mental and physical health (Ranson & Urichuk, 2008). Insecure PCA may increase the risk for later behavior problems, psychopathology, and physical illnesses, especially in the presence of other risk factors, such as poverty or abuse (Anderson et al., 2012; Cassidy et al., 2014; Puig et al., 2012; Sroufe et al., 1999).

The discipline of nursing has a strong interest in promoting child health (Fuemmeler et al., 2017). Nurses work closely with families of young children, and parents often approach nurses for advice on their children’s health (Ordway et al., 2014). Thus, nurses have a unique opportunity to promote secure PCA, especially among families where children are at increased risk for developing insecure attachments (e.g., families affected by poverty, intimate partner violence, stress, parental mental health problems, and substance use). However, descriptions of the PCA concept have been inconsistent in nursing literature, slowing the advancement of nursing practice in this area. For example, a previous concept analysis on maternal-infant attachment (Goulet et al., 1998) and practice guidelines (Schenck et al., 2005) included several statements that described maternal-infant bonding, thus confusing the distinction between attachment and bonding in the nursing literature. We assert that nursing practice will be enhanced by a clearer understanding of attachment theory and that nurses will be better positioned to support parents, increasing the likelihood of secure attachment development, and subsequently, improving long-term child’s health outcomes.

## Aims of Analysis

We aimed to review how PCA is described across the disciplines of psychology and nursing to advance the use of this concept for nursing practice. A multidisciplinary perspective is important for nursing because other disciplines can add to the understanding of a concept of interest (McCrae, 2012). While the concept of PCA originated in the discipline of psychology, a clear definition and careful use of terminology are critical for this concept to be successfully adopted in nursing practice.

## Method

**Figure 1.**
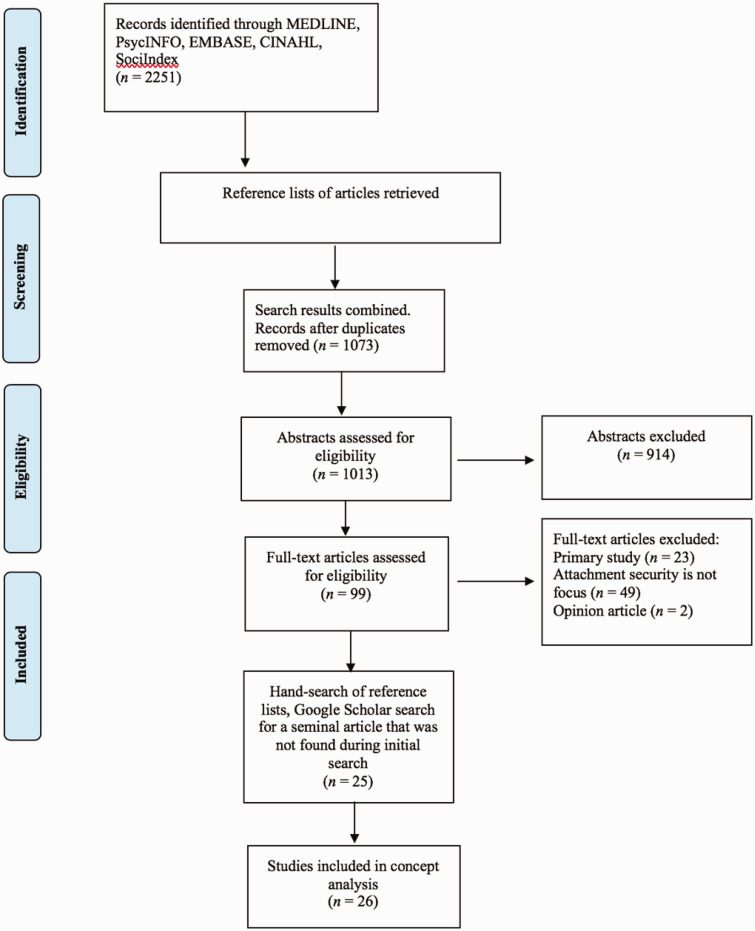
PRISMA 2009 Flow Diagram (Moher et al., 2009).

Literature searches were conducted in MEDLINE, EMBASE, CINAHL, ERIC, and PsycINFO databases using combination sets of keywords (1: child; 2: parent; 3: attachment) in titles and abstracts of the articles (Table 1). Search terms were pilot-tested with an academic librarian at the University of Calgary and refined for each database. No time limits were set. The literature searches were conducted following the Preferred Reporting Items for Systematic Reviews and Meta-Analyses (PRISMA) guidelines (Figure 1, Moher et al., 2009). Articles were included if they met the following criteria: (1) published in English, (2) literature reviews or conceptual papers, (3) included children ≤ 18 years of age and their parents (mother or father), and (4) parent-child attachment identified as a focus. Articles were excluded if they were (1) primary studies, (2) focused on parental-fetal attachment, (3) focused on attachment disorders, and (4) editorials, letters, books, abstracts, and grey literature (Table 2). Reference lists of publications were examined for further potentially relevant sources. A data file using an Excel spreadsheet was created to aid in the screening and synthesis of the literature. The data file captured the following: authors’ names, publication year, authors’ field of discipline, abstract, PCA definition, children’s age in the publication, attachment to mother/father/both parents.

**Table 1.** Search Strategy Key Words.

Database	Search strategy key words
OVID databases	((father* or paternal* or mother* or maternal* or parent*) adj2 child* adj2 attachment*).mp.
EBSCO databases	(father* or paternal* or mother* or maternal* or parent*) N2 child* N2 attachment

**Table 2.** Inclusion/Exclusion Criteria.

PICO	Inclusion criteria	Exclusion criteria
Population	Children aged ≤ 18 years Parents (mother and/or father) of children aged ≤ 18 years	Fetus Parent-fetus relationship Animals
Concept	Parent (mother/father)-child (infant) attachment Attachment as defined by Bowlby and Ainsworth, a relationship between a primary caregiver (attachment figure) and a child where a child uses an attachment figure as a safe haven in times of distress and a secure base from which to explore	Parent-fetal attachment Attachment disorders Parent-child bonding Parent-child attachment in adulthood
Article type	Literature reviews Discussion or conceptual papers	Primary studies Animal studies Commentaries, opinion articles, books, book reviews, conference abstracts, dissertations

## Results

Two reviewers (first author and the scientific writer/methodologist at the Faculty of Nursing, University of Calgary) conducted an inter-rater reliability check (McHugh, 2012) with 97% agreement, and independently screened 1013 abstracts. Ninety-nine abstracts were selected for full-text review. A total of 26 articles were identified as meeting the inclusion criteria of this analysis. The majority of included articles were from the discipline of psychology (*n* = 22), three were from the discipline of nursing, and one article was from the medical discipline of medicine. Most of the articles were written in North America (*n* = 20). Four articles focused on father-child attachment, while the rest examined either mother-child attachment (*n* = 8), attachment between both parents and a child (*n* = 5), or did not specify which parent was the focus (*n* = 9) (Table 3). Using the principle-based concept analysis method (Morse, 1995; Penrod & Hupcey, 2005), the articles were examined to clarify the definition of the PCA concept (epistemological principle), appraising its applicability in practice (pragmatic principle), evaluating consistency in use within literature (linguistic principle), and differentiating it from related concepts (logical principle). See Table 4 for the description of the application of guiding principles to the analysis.

**Table 3.** Included Studies.

First author, year	Country	Child age	Type of article	Aim	Parent (mother/father)	Definition of attachment used	Discipline
Atkinson, 2000	Canada	≤ 36 months	Meta-analysis	To address association between attachment security and maternal social-marital support, stress, and depression.	Mothers	Not provided	Psychology
Ainsworth, 1979	USA	≤ 12 months	Conceptual	To describe development and use of Strange Situation Procedure	Mothers	“babies use their mothers as a secure base from which to explore…, their attachment behavior is greatly intensified by the separation…they seek contact with, proximity to, or at least interaction with their mothers”	Psychology
Benoit, 2004	Canada	Not specified	Conceptual	Review pertinent aspects of attachment theory and research	Not specified	“One specific and circumscribed aspect of the relationship between a child and caregiver that is involved in making the child safe, secure and protected”	Psychology
Bretherton, 2010	USA	Not specified	Qualitative review	To provide history of attachment research on fathers	Fathers	“Mantains a younger more vulnurable individual in more or less proximity to another discriminated and stronger individual who can provide protection”	Psychology
Bosman, 2016	USA	Young children and adolescents	Qualitative review	To dicuss componets of CBT, identify conceptual problems with attachment theory, review research that supports use of CBT in restoring attachment relationships	Not specified	“…children can use the caregiver as a *secure base* from which they can freely explore their environment and…a *safe haven* to turn to when they need support during distress”	Psychology
Feeney, 2000	Australia	Not specified	Conceptual	To describe how chilren with different attachment styles respond to pain and physical illness	Not specified	“the bonds of affection that develop between children and their caregivers (usually the parents)…these bonds reflect humans’ universal needs for comfort and security”	Psychology
Feiring, 1984	USA	Infancy	Conceptual	To dicuss developmental path from from early modes of behavior to later adult personality styles as continuum of adaptive functioning	Not specified	“confidence in the availability and responsiveness of one or few adults and the ability to use these adults as a secure base from which to explore the environment”	Psychology
Flaherty, 2011	USA	Infancy	Conceptual	Review attachment theory and relate attachment perspective to adolescent mothers and their children	Mothers	“attachment provides a secure base from which the child can explore the world…a unique relationship between an infant and his or her caregiver that is foundation for further healthy development”	Nursing
George, 1996	USA	Infancy and middle childhood	Conceptual	To examine role of IWMs in the development of child-parent relationships	Not specified	“an instinctive reciprocal relationship…primary function of this relationship is *protection* of the child”	Psychology
Goulet, 1998	Canada	Infancy	Concept analysis	To define the attributes, antecedents, and consequences of the concept of parent-infant attachment, to refine measurements of concept	Not specified	“a link between two people”	Nursing
John, 2010	USA	Not specified	Conceptual	Highlight relevance of Stephenson’s Q methodology for improving the assessment of child-father attachment	Fathers	“the child’s use of the mother as a ‘secure base’ to explore from and as ‘secure haven’ to seek comfort during distress”	Psychology
Koehn, 2018	USA	5-18 years of age	Meta-analysis	To examine relationship between parent-child attachment and multiple components of parenting in children 5-18 years of age	Not specified	“relationship that forms between a child and primary caregiver…children form bonds…to elicit support and comfort”	Psychology
Kindsvatter, 2018	USA	Not specified	Review	To examine the developmental trajectory of children with attachment disorganization across the life span	Not specified	“Emotional bonds in early life”	Psychology
Letourneau, 2015	Canada	≤ 12 months	Narrative systematic review and meta-analysis	To examine effectiveness of interventions aimed at promoting maternal sensitivity and reflective function on maternal-child attachment security	Mothers	“affective quality and organization of the relational transactions between a caregiver (most often the mother) and child”	Nursing/Psychology
Lyons-Ruth, 1996	USA	Not specified	Conceptual	To review attachment-related studies of early aggression	Not specified	“does not refer to all aspects of the parent-child relationship…has a goal of a reduction of arousal and reinstatment of a sense of security, usually achived in infancy by close physical contact with a familiar caregiver”	Psychology
Palm, 2014	USA	Not specified	Qualitative review	To review attachment theory as related to father-child relationships during early childhood years	Fathers	“an intense and enduring emotional bond that is rooted in the function of protection of infants from danger…provides asecure base for the child to explore and a safe haven to return to in times of distress”	Psychology
Paquette, 2004	Canada	Not specified	Conceptual	To propose a theorization of the father-child relationship based on current understanding of attachment, interactions between fathers and their young children, and human-specific adaptations	Fathers	“emotional bond between a mother and her child promotes physical proximity between the two thus ensuring the care and protection of the child”	Psychology
Posada, 2013	USA	Preschoolers	Review	To review origins of the sensitivity constructs highlighting the cross-cultural lens of Ainsworth’s research in Uganda and Baltimore and the role played by the methodology she used	Both	Not provided	Psychology
O’Connor, 2000	United Kingdom	Not specified	Review	To consider how methods and theories of behavioral genetics and attachment theory are mutually informative	Both	Not provided	Psychology
Ranson, 2008	Canada	12 months -18 years of age	Review	To review the impact parent-infant attachment relationship may have on a variety of biopsychosocial domains, across developmental periods	Both	“A significant aspect of the parent-child relationship”	Medicine
Rosmalen, 2014,	The Netherlands	Not specified	Review	To add a new perspective to the histography of the SSP	Mothers	“Human infants need a consistent nurturing relationship with one or more sensitive caregivers in order to develop into healthy individuals”	Psychology
Sroufe, 1977	USA	12-24 months	Conceptual paper	To argue for the importance of understanding of attachment behaviors from organizational perspective, to critique research where attachment behaviors are taken outside of context	Mothers/caregvers	“An affective tie between infant and caregiver…behavioral system, flexibly operating in terms of set goals, mediated by feeling”	Psychology
Van Ijzendoorn, 1992	The Netherlands/Canada	Infants	Meta-analysis	To test whether maternal problems lead to more deviating attachment classification distributions than child problems	Mothers	“Species-specific behaviors in infants that are effective in eliciting caregiver proximity and protection as well as reciprocal species-specific behaviors in adults”	Psychology
Van Ijzendoorn, 1995	The Netherlands	0-18 months	Meta-analysis	To address effectiveness of preventative or therapeutic interventions aiming at enchancing parental sensitivity and children’s attachment security	Mothers	Not provided	Psychology
Verhage, 2018	The Netherlands	11-96 months	Meta-analysis	To examine whether ecological factors may explain variability in the strength of intergenerational transmission of attachment	Both	“a set of conscious and/or unconscious rules for the organization of information relevant to attachment and for obtaining or limiting access to that information”	Psychology
Zeanah, 2011	USA	Infants and young children	Review	To summarize issues from attachment theory and research and discuss how these issues inform clinical work with infants and young children	Both	“A strong disposition to seek proximity to and contact with a specific figure and to do so in certain situations, notably when frightened, tired or ill” “The infant’s or young child’s emotional connection to an adult caregiver-an attachment figure-as inferred from the child’s tendency to turn selectively to that adult to increase proximity when needing comfort, support, nurturance or protection”	Psychology

**Table 4.** Guiding Principles (Penrod & Hupcey, 2013) and Application to Concept of PCA.

Principle	Definition	Application
Epistomological	“Focuses on the discipline’s distinction of concept within the knowledge base” (p.405).	Is concept clearly defined in nursing and related literature; how well is it differentiated from other concepts.
Pragmatic	“applicability to explaining or describing phenomeonon encountered within discipline…from the perspective of usefulness” (p. 405).	Describe how concept of PCA is defined in literature. Is it usefull to the discipline of nursing?
Linguistic	“evaluates the approapriate use of concept…consistency in use and meaning are considered. Concepts should be appropriate to their use in context” (p. 406).	Is PCA concept used consistently and approapriately within the literature?
Logical	“Integration of the concept with related concepts” (p. 406).	How well does PCA concept holds its boundaries when integrated with related concepts?

### Epistemological Principle: Is the Concept Clearly Defined?

The PCA is a “psychological tether which binds infant and caregiver together” (L. Sroufe & Waters, 1977, p. 1186). Bowlby (1951) argued that to thrive emotionally and grow up mentally healthy, a child must experience a mutually affectionate relationship with the primary caregiver (attachment figure). When distressed or alarmed, children are disposed to attain closeness with their attachment figure through cues and movements, such as crying, smiling, sucking, clinging, and crawling. This disposition towards an attachment figure is described as an attachment system, with the actions of the child being termed attachment behaviors (Bowlby, 1969/1982). Attachment behaviors are influenced by situational context (e.g., setting, preceding events) and the child’s mood, and are not constant across situations (L. Sroufe & Waters, 1977). Attachment behaviors are differentiated from affiliative behaviors because they involve seeking proximity when experiencing distress (Zeanah et al., 2011). Reciprocity of the behaviors between caregiver and child is the key factor in Bowlby and Ainsworth’s descriptions of attachment. Bowlby (1969) described attachment figure-child proximity as a set- goal of the attachment system, with attachment behaviors (smiling, clinging) serving to achieve this goal. Attachment behaviors change as children mature. For example, infants who seek proximity by crawling towards the caregiver at 12 months of age may smile and speak when they are older. Thus, multiple attachment behaviors can have a similar meaning, and serve as means of re-establishing contact (Sroufe & Waters, 1977). The child decides which behaviors to use to maintain the proximity to the caregiver based on the situational context (Sroufe & Waters, 1977). Sroufe and Waters (1977) suggested that the set- goal of the attachment system is “felt security”, where the child feels affection for the caregiver. There is a decreased need for proximity to the attachment figure in the absence of distress as the child grows and develops.

Two hallmark ideas of attachment theory are “secure base” and “internal working models”. The concept of the secure base contains two interconnected phenomena: a “base” from which to explore and a “haven of safety” to which the child may return in times of distress (Waters & Cummings, 2000). The attachment figure for the child is regarded as a "secure base", a stronger and wiser person to turn to when the attachment system is activated (such as when a child is frightened or ill). Once comfort and reassurance in the presence of distress-inducing stimuli are achieved, the attachment figure “shifts from being a safe haven to being a secure base from which to explore” (Ainsworth et al., 1978, p. 265). The feeling of security promotes the child’s return to play (Sroufe & Waters, 1977). Bowlby (1988) proposed that across the lifespan “all of us…are happiest when life is organized as a series of excursions, long or short, from the secure base provided by our attachment figure” (p. 62). A child’s committed relationship with at least one attachment figure is central to healthy development (Bretherton, 1992). An attachment figure is someone who provides “physical and emotional care, continuity and consistency in a child’s life, and emotional investment in the child” (Howes, 1999, p. 675). Bowlby used the term “attachment figure” rather than “mother” presuming that the nature of the interaction rather than the category of the individual (e.g., adoptive mothers, siblings, grandparents, fathers, childcare providers) would be of most importance to the child (Ainsworth et al., 2015). Sir Richard Bowlby (2010) suggested that children can have a network of multiple attachment figures. All four attachment patterns are relationship-specific, such that a child may show secure attachment towards the mother, be avoidantly attached to the father, and display disorganized attachment behaviors towards the daycare worker.

The attachment figure needs to be accessible and available to the child, along with being perceptive of the child's emotional needs. Parents function as attachment figures to children across the early years, preadolescence, and adolescence (Kerns & Brumariu, 2014). As children grow, they venture further from the secure base, and for an increasingly extended period of time (Bowlby, 1988). Thus, a parent's behavior is expected to shift from a protective role in the early years of a child's life to autonomy-support and encouragement of self-regulation as the child gets older (Koehn & Kerns, 2018).

The responsiveness of the attachment figure to the child’s signals of a need to explore is as important as the sensitivity to signals of proximity needs (Grossmann et al., 2002). Children trust that their attachment figures will notice and respond to their needs (Benoit, 2004). This trust facilitates the exploration of the environment and supports the development of social and cognitive competence (Ainsworth et al., 2015). Ainsworth used the term “organization” to describe the way a child’s attachment behaviors are brought together to form a coherent pattern of security or insecurity (Duschinsky & Solomon, 2017). Quality of PCA is best assessed by reference to organization of attachment behaviors concerning the attachment figure and in consideration of the situational context (L. Sroufe & Waters, 1977). Children with secure attachment (Type B) openly express their emotional needs and can accurately predict responses from their caregivers. Children with insecure/avoidant attachment (Type A) mask their emotional needs, typically after being consistently rejected by their caregivers in times of past distress. In insecure/ambivalent (Type C) attachment relationships, children exaggerate the display of their emotional needs in a desperate attempt to get the attention of the caregiver, who has typically demonstrated inconsistency in their past responses. Children with secure and insecure/avoidant and insecure/ambivalent patterns use organized strategies to maintain interaction with their attachment figure, such that children *know* how to deal with distress ful situations to maintain a satisfying interaction with their attachment figure (Benoit, 2004). Children with insecure/disorganized (D) attachment, fail to develop and organized pattern and display contradictory and incoherent behaviors with caregivers. Secure attachment is the most prevalent and preferred pattern of PCA across cultures. See Table 5 for the description of patterns of attachment.

**Table 5.** Patterns of Attachment, Distribution, and Corresponding Responses.

Caregiver behaviors	Child’s attachment strategy	Child’s reactions and expectations	Attachment patterns	Distribution in low-risk populations
Caring, loving	Organized, seeks proximity and maintains contact until soothed, able to return to play and exploration	Trust in others, trust in self: “you are good and I am good”.	B	Approximately 65%
Rejecting, unresponsive, unavailable	Organized, avoids caregiver when distressed, inhibit emotional arousal in the presence of the caregiver	Trust in self, distrust in others: “I will do this on my own, I fear closeness”.	A	Approximately 35%
Overinvolved or inconsistent	Organized, extreme displays of negative emotions and/or helplessness	Needy for others: “I am dependent on you and I fear abandonment”.	C	Approximately 15%
Dissociated, frightening, anomalous or frightened	Disorganized	Frightened of self and others: “You are scary, I am scary” Unescapable fear originating from within. Inability to assess safe haven.	D	Approximately 15% (up to 85% in high-risk groups)

*Note.* Adapted from Benoit (2004) and Golding (2007).

Another key idea of attachment theory, internal working models (IWMs), is defined as cognitive mental structures or mental schemas. IWMs summarize early caregiving experiences and shape expectations about future interactions with significant others (Bretherton, 1992). IWMs are thought to develop as early as 12 months of age, constructed from a child’s actual experiences with the parent (George, 1996), and influence how the children view themselves in relationships with others (Rosmalen et al., 2014). The concept of IWMs has received much attention in explaining the link between early caregiving experiences and children’s later health outcomes. For example, securely attached children develop IWMs of available care and self-worth and tend to be more empathetic and responsive to others (Sroufe et al., 1993). Children with insecure attachments develop IWMs of the self as unworthy and expectations of others as unavailable or insensitive. These children are more likely to inadequately cope with stress and behave in ways that bring about more adverse experiences (Sroufe et al., 1993). As children grow, they begin to rely more on IWMs than on the actual presence of the attachment figure to guide their daily social interactions (Bowlby, 1982).

To conclude, the concept of PCA is well-defined within the psychology literature. Most articles in this review employed Ainsworth et al. (1978) and/or Bowlby’s (1988) definitions of PCA (see Table 3), with an exception of a nursing article by Goulet (1998), where the definition of PCA by Bowlby (1996) was mixed with the definition of maternal-infant bonding by Klaus and Kennell (1983). The definitions of PCA in the nursing literature were mostly focused on infants, and not on older children.

### Pragmatic Principle: Is the Concept Applicable and Useful?

An extensive amount of literature focused on the measurement of attachment in infancy and childhood. The development of a standardized procedure to observe and study attachment behavior was a major step in the establishment of an empirical knowledge base regarding the developmental significance of attachment (Ainsworth et al., 1978). A study of individual differences in child attachment in home- and laboratory-based observations in 26 middle-class families in Baltimore led to the development of the Strange Situation procedure (SSP) (Ainsworth et al., 1978; Bell & Ainsworth, 1972) to classify patterns of attachment. The SSP was designed to use cues of unfamiliarity and separation from a caregiver to elicit attachment behaviors. The SSP aimed to activate the child's expectations about what happens when the caregiver was not available in the past and allowed the observer to interpret these expectations from the child’s behavior (Duschinsky, 2015). The first report on the use of SSP described 14 children from the Baltimore study who were divided into three groups based on their behavior during the procedure (Ainsworth & Bell, 1970). The SSP consists of eight episodes (see Table 6). During SSP, a caregiver leaves the child twice in an unfamiliar environment (a laboratory room decorated as a playroom) with enticing toys, first with a stranger (an unknown female experimenter) and then alone, before returning (Ainsworth et al., 1978). Being separated twice in an unfamiliar (*strange*) environment activates the child’s attachment system and allows for observation of individual differences in attachment patterns (Rosmalen et al., 2014). The decisive factor for attachment classification is the child's behavior at the reunion with the caregiver. The SSP is videotaped and the child's behaviors concerning the caregiver are coded on a 7-point Likert scale for the presence of proximity and contact-seeking, maintaining contact, resistance, avoidance, distance interaction, and search behaviors (Shah & Strathearn, 2014).

**Table 6.** The Strange Situation Procedure Episodes (Ainsworth et al., 1978; Rosemalen et al., 2014).

1. The caregiver and infant enter the room (30 seconds)
2. The caregiver and infant are in the room (3 minutes)
3. A stranger enters (3 minutes)
4. The caregiver leaves the room. The stranger and caregiver are in the room (3 minutes or less)
5. The caregiver returns and stranger leaves. Caregiver leaves again (3 minutes or less)
6. The infant is alone in the room (3 minutes or less)
7. The stranger returns (3 minutes or less)
8. The caregiver returns and stranger leaves (3 minutes or more)

In 1986, Main and Solomon reviewed 200 cases from the early SSP tapes and added a fourth category (D) to describe a child’s inexplicable and fragmentary behaviors that were discrepant with the original ABC classification. A child’s behaviors coded as D were based on the following indices: (a) sequential and (b) simultaneous display of contradictory behavior, (c) misdirected or incomplete movements, (d) stereotypes and anomalous postures, (e) freezing or stilling, (f) display of apprehension of the caregiver, and (g) signs of disorganization (Granqvist et al., 2017). A 9-point scale for coding disorganization is used, where observation of five or more disorganized behaviors is sufficient to place a child into the D category (Duschinsky & Solomon, 2017). Combined with Ainsworth’s original attachment patterns, this discovery culminated in the ABC+D attachment classification model. The SSP remains one of the most widely used standardized, laboratory-based assessments of young children's attachment behaviors (Fearon et al., 2010).

The SSP is normally only used during infancy and toddlerhood because as the children grow, the goals of attachment shift from proximity-seeking to co-constructing of cooperative partnerships with their attachment figure to ensure parents' ability and availability to act in child's best interests (Fernandes et al., 2020). To account for the change in the attachment goal and behaviors, different measures of PCA are used during middle childhood and adolescence. Some of the examples of these measures include Maternal Behavior Q-Set (Pederson & Moran, 1995), Attachment Story Completion Task (Bretherton et al., 1990), Adult/Adolescent Attachment Interview (George et al., 1996), Security Scale (Kerns et al., 2001), and separation-reunion procedures (Solomon & George, 1999).

The SSP is validated against home observations of mother-child interactions, and some suggest that it may not capture the quality of attachment to fathers (Bretherton, 2010; Grossmann et al., 2002). The mechanisms involved in the development of attachment relationships may differ for mothers and fathers, with a mother’s interactions with her child typically involving caregiving, while a father’s interactions typically involve play (Lamb, 2000). Paquette (2004) proposed a new theory of father-child attachment, called activation relationship. The activation relationship theory stipulates that limit-setting and discipline are important in helping children feel safe when exploring their environment (Paquette, 2004). The Risky Situation (RS) procedure has been developed to test the following dimensions of father-child attachment in children aged 12-18 months: stimulation (encourage the child to be open to the outside world), and discipline (setting limits for the child’s safety). According to the activation relationship theory, children are classified as activated (explore environment appropriately, and obey limits set by the father for their protection), under activated (fearful to explore, cautious and obedient), or overactivated (showing no hesitation or fear in exploration, even in the presence of risk, and are disobedient) (Paquette, 2004).

One of the central notions of attachment theory is that the quality of early caregiving is crucial to child development, especially how the parent is sensitive and responsive to the child’s cues (Ainsworth et al., 1978; Bell & Ainsworth, 1972). Attachment-based interventions are usually aimed at helping parents become more sensitive, responsive, and reflective. Sensitivity is the parental ability to perceive and appropriately interpret the cues implicit in the child's behavior, verbalization, and words, and correctly respond to these signals (Ainsworth et al., 1978). Individual differences in the child’s attachment security were attributed to variations in maternal sensitivity, and mothers of securely attached children were deemed more consistent and sensitive than mothers of insecurely attached children (De Wolff & van Ijzendoorn, 1997). Numerous studies have replicated these findings, exploring how maternal capacity to organize thoughts was linked to the ability to sensitively respond to her infant (van Ijzendoorn, 1995). Reviews by Goldsmith and Alansky (1987), van Ijzendoorn (1995), De Wolff and van Ijzendoorn (1997), and Madigan et al. (2006) supported the notion that maternal sensitivity was linked to attachment security. A review of interventions to increase infant security by enhancing parental sensitivity concluded that short-term interventions were more effective than long-term, and increase in parental sensitivity did not always impact an infant’s attachment security, and in some studies improvement in infant security was noted without an increase in parental sensitivity (Mountain et al., 2017).

The security of a child's attachment is also associated with the parent’s reflective functioning (RF) (Katznelson, 2014; Slade, 2005). Parental RF refers to a parent's capacity to reflect on their own childhood experiences, and make meaning of their child’s thoughts, feelings, and behaviors (Slade, 2005). It is theorized that RF promotes the parental capacity to regulate their emotions, and enables them to respond sensitively to their children’s emotional and physical needs (Fonagy & Target, 1997). Higher levels of maternal (Stacks et al., 2014) and paternal (Fonagy et al., 1991) RF have been linked to sensitive behavior and a child’s attachment security. Studies that have assessed maternal reflective functioning capacity have shown that mothers with moderate to high RF scores were more likely to respond to their children sensitively, and were more likely to have children with secure attachment, compared to mothers with lower RF scores (Bakermans-Kranenburg et al., 2003; Sadler et al., 2013).

To conclude, the concept of PCA can be measured by validated tools and is useful to nursing practice because nursing practice is concerned with child mental health (Gordon et al., 2020).

### Linguistic Principle: Is the Concept Used Consistently and Appropriately Within the Scientific Literature?

The most outstanding linguistic issue pertained to how PCA differs from maternal-child bonding. In the nursing literature, PCA is often conflated with parent-child bonding (Bicking Kinsey & Hupcey, 2013; Klaus & Kennell, 1983). While screening abstracts to determine inclusion eligibility for this concept analysis, we noted that many nursing articles that included the term “attachment” in the title and/or abstract used the terms “attachment” and “bonding” interchangeably. Maternal-infant bonding is emotional affection experienced by the mother toward her infant emerging in the immediate postpartum period (Condon & Corkindale, 1998). Bonding is not attachment (Benoit, 2004; Redshaw & Martin, 2013). Unlike PCA, bonding does not predict socio-emotional and physical health outcomes later in life. Blurred distinctions between the concepts of bonding and PCA and the subsequent practice recommendations from nursing articles (Goulet et al., 1998; Hill & Flanagan, 2020; Schenk et al., 2005) suggest that a clarification of PCA concept is needed.

There is a discrepancy in the description of the patterns of attachment within the psychology and nursing literature, indicating that nurses and psychologists may think differently about PCA. The conventional terms (developed by Bowlby & Ainsworth) used in the psychology literature for quality of attachment are “secure”, “insecure”, or “disorganized”. In the nursing literature, terms such as “positive” or “negative” are used when referring to the quality of PCA (Schenk et al., 2005). Further, the terms “attachment” and “bonding” are used interchangeably in nursing original research and conceptual articles (Karl et al., 2006; Kearvell & Grant, 2010; Zauderer, 2008). From the nursing perspective, attachment begins immediately after birth (do Vale et al., 2006; Goulet et al., 1998; Schenk et al., 2005). For example, a descriptive nursing study suggested that “attachment initially occurs soon after birth in a very important manner through eye contact” (do Vale et al., 2005, p. 71). From the psychology perspective, this statement is problematic, because attachment is not an instantaneous process, and only develops by the age of six or eight months (Zeanah et al., 2000).

The scales frequently used by nurses to assess bonding have the term “attachment” in the title, such as the Maternal-Postnatal Attachment Scale (Beirami et al., 2017; Ghadery-Sefat et al., 2016), which may also contribute to differences in how PCA is understood in the nursing and psychology literature.

Schenk et al. (2005) claimed that the nature of maternal-child attachment “from birth” (p. 516) is associated with the child’s behavioral disorders. The latest research suggests that maternal mental health problems and perception of family support, not the quality of bonding at birth, may influence the development of a child's behavior problems (Maia et al., 2020). The discrepancy between how bonding and attachment are understood is not just a “harmless misunderstanding” (Eyer, 1994, p. 90). New parents who have been separated from their newborn often feel anxiety about not being able to establish “bonding” early on (Redshaw & Martin, 2013), but bonding has not been shown to impact long term emotional and physical health.

In sum, PCA is not bonding. The implied meaning of the PCA concept in nursing literature is inconsistent with that of psychology and medicine. PCA does impact health and development over the life span (Puig et al., 2013; Sroufe, 2005; Weinfield et al., 2000). Claims regarding the effects of maternal-infant bonding on long-term child health outcomes are not supported by current evidence.

### Logical Principle: Does the Concept Hold Its Boundaries When Integrated With Other Concepts?

In applying the logical principle, we considered PCA in the context of related concepts, such as “parenting” and “parent-child relationship”. There is often an overlap between concepts of “attachment” and “parenting” in the scientific literature (Koehn & Kerns, 2018). There is much more to parenting than attachment (Lyons-Ruth, 1996). Attachment is only one specific aspect of a relationship between a parent and a child. The purpose of an attachment figure is to make the child feel safe, secure, and protected. Attachment is different from other aspects of parenting such as feeding, teaching, playing, and disciplining (Benoit, 2004). However, attachment-based research has been used to identify parenting practices that can impact a child's socio-emotional health, such as frightening or frightened parental behavior that may contribute to attachment disorganization (Kindsvatter & Tansey, 2018; Lyons-Ruth, 1996; Ranson & Urichuk, 2008).

The parent-child relationship is another concept that frequently emerged during our review of the literature for this concept analysis. The relationship is defined as the state of being related or interrelated (Merriam-Webster Dictionary, 2019). Parent-child relationships influence the social and emotional development of the child (Koehn & Kerns, 2018). Nonetheless, PCA is not the same as the concept of a parent-child relationship. Rather, attachment theory provides a framework for studying parent-child relationships (Van IJzendoorn, Goldberg et al., 1992). Attachment is a specific type of relationship with distinct goals and behaviors. Additionally, PCA can function outside of parent-child relationships, as in cases of securely attached children shown to have less difficulty in building and maintaining social relationships with peers (Sroufe, 1988). To conclude, there are conceptually distinct boundaries between PCA, parenting, and the parent-child relationship.

## Recommendations for Nursing Practice

The concept of PCA is useful and relevant to nursing practice. A child's mental health is one of the top national priorities for nursing (Eckardt et al., 2017). The nurse may be the first healthcare professional who comes in contact with parents and their children. Nurses in all areas of healthcare should be able to observe the interaction between a parent and a child (e.g., attachment behaviors), and assess the quality of PCA. Nurse leaders must continue to advocate for the inclusion of attachment theory knowledge in formal nursing programs to help ensure the implementation and sustainability of attachment-based interventions that support optimal child development. Nurses need to identify factors that interfere with PCA, assess available supports, and provide necessary referrals. Specifically, the assessment of the PCA should include an assessment of the family context (e.g., parental mental health, social support, socio-economic status) because children are highly dependent on their immediate family environment for their health and development.

Even maltreated and neglected children become attached, although usually in an insecure or disorganized way (Benoit, 2004). When assessing PCA, the question should be *what is the quality* of PCA instead of *is there* a PCA (Benoit, 2004). PCA can be disrupted by conditions that limit children’s behavior, such as neurological disorders, illness, and fatigue, or conditions that can interfere with the parent’s ability to provide care, such as poverty and mental health problems. However, the parent plays a more important role than the child in shaping the quality of attachment (O'Connor et al., 2019). This is important to know because attachment outcomes can be altered by changes in the immediate environment, such as changes in parenting behaviors (Belsky & Fearon, 2002).

Attachment behaviors are most likely to be evident when the child is in an unfamiliar environment or feeling ill or in pain (Feeney, 2000). During the visit to a healthcare setting, a frightened child may crawl to the parent, looking to be picked up and comforted (George, 1996). Nurses can point out the child's attachment behaviors, such as clinging to the parent when upset, resisting separation, and calming when held by the parent. In toddlers, controlling behaviors (e.g., being punitive, bossy, assuming inappropriate caregiving behaviors towards a parent) can be noted as possible signs of attachment insecurity/disorganization (Zeanah et al., 2011). It is important to note that a secure or insecure attachment quality cannot be inferred from any single behavior (e.g., crying, resistance), but needs to be inferred from the pattern of behavior within situational context (separating or reuniting with a parent) (Sroufe & Waters, 1977). Parenting behaviors that encourage autonomy and maintain relatedness (e.g., respecting that children have their own thoughts and feelings) are important indicators of attachment security in parents with adolescents. To enhance PAC, nurses can use positive reinforcement of parenting skills, such as modeling appropriate responses (e.g., warm, supportive, sensitive, responsive, and prompt) to the child’s behaviors to encourage parents to assume similar responses to promote feelings of security in a child. Nurses should provide education on ways to increase parental sensitivity and responsiveness (child cue-based interactions, normal child growth, and development).

Both mother and father are important in fostering secure attachment (Bretherton, 2010). Fathers are still marginal to the majority of parenting interventions to enhance PCA (Panter-Brick et al., 2014), and more research that evaluates parenting programs that include the participation of fathers is needed to better understand the impact of fathering on a child's attachment security. Because attachment-based intervention programs have primarily targeted the mother-child relationship, there are no guidelines for effective father-child attachment programs to date (Henry et al., 2020). Adverse socio-demographic factors have been shown to have a more negative impact on the fathering role, compared to the mothering role (Bretherton, 2010; Palm, 2014; Paquette, 2004). Thus, the most effective programs to support fathers should include efforts to access and minimize socio-demographic barriers (e.g., lack of social support and unemployment, marital discord, mental illness, lack of education) along with parenting skills (e.g., sensitivity, responsiveness towards their children) and knowledge of child development (Bretherton, 2010; Palm, 2014). With the support of nurses, fathers can develop parenting competencies and confidence in both play and caregiving contexts which can enhance father-child attachment security.

To advance the use of the PCA concept for nursing practice, we propose the definition of the PCA as a bi-directional relationship between a child of 0 to 18 years of age and a primary caregiver, where a child uses the caregiver as a secure base from which to explore and as a haven of safety and source of comfort in times of distress (Ainsworth et al., 1978; Bowlby, 1988).

## Conclusions

This article aimed to describe how the concept of PCA is understood from a multidisciplinary perspective. Attachment theory offers nurses promising practice opportunities to provide an umbrella of supports that embraces and promotes life-long emotional and physical health of children and their families. The concept of PCA is poorly understood in nursing literature due to inconsistencies and confusion of this concept with the concept of bonding.
